# Multimode Input Enhancement of Absorption Sensing of Methane in a Hollow Bottle Microresonator

**DOI:** 10.3390/s25175458

**Published:** 2025-09-03

**Authors:** Mohmad Junaid Ul Haq, A. T. Rosenberger

**Affiliations:** Department of Physics, Oklahoma State University, Stillwater, OK 74078-3072, USA; mjunaid@okstate.edu

**Keywords:** microresonator, whispering-gallery modes, dissipative sensing enhancement, multimode fiber

## Abstract

Dissipative sensing in a whispering-gallery-mode (WGM) microresonator entails monitoring changes in WGM throughput dip depth or linewidth due to analyte absorption. In our earlier work, we showed that dip depth sensitivity can be two orders of magnitude greater than linewidth sensitivity for sensing the broadband absorption of a dye in methanol. Here we experimentally demonstrate enhancement of absorption sensing of methane. Its narrowband absorption lines (a few GHz linewidth) necessitate strain tuning of the WGM of our hollow bottle resonator (HBR) to bring the WGM into resonance with the absorption line. Three asymmetric tapered fibers with different nonadiabaticities were designed to excite multiple fiber modes that couple into the WGM to interact with methane inside the HBR via the internal evanescent field. Measurements were carried out for both pure and trace (in 1 atm of air) methane at 1654 and 1651 nm. Enhancement factors as large as 141 were found; the experimental results agree with theoretical calculations and with the predictions of a limiting-case model. Effective absorption path lengths as large as 273 cm, more than ten thousand times the HBR diameter, were achieved for trace methane sensing, with detection limits estimated to be in the hundreds of ppm.

## 1. Introduction

Whispering-gallery-mode (WGM) microresonators have emerged as highly sensitive platforms for detecting physical, chemical and biological changes in the surrounding medium [1,2,3,4,5]. These sensors operate by monitoring perturbations in the fiber-coupled throughput spectra and can be classified as dispersive or dissipative based on the sensing mechanism. Dispersive sensing relies on shifts in the resonance frequency of the WGM and has been studied to detect refractive index changes, molecular adsorption, and environmental factors such as temperature, pressure, and force. Dissipative sensing is based on changes in the spectral characteristics of the WGM, specifically linewidth and throughput dip depth, due to loss modifications caused by absorption or scattering in the presence of an analyte. Previous works have shown that in an adiabatically tapered fiber-coupled microresonator system (single-mode input), operating in the overcoupled limit, dissipative sensing based on dip depth change can be more sensitive than both dispersive sensing based on frequency change and dissipative sensing based on linewidth change [6,7]. Overcoupling is not always easy to achieve, but we recently showed that an effective strong overcoupling can be synthesized by using an asymmetric tapered fiber, having a nonadiabatic downtaper and an adiabatic uptaper, to produce two-mode input [8]. Using such nonadiabatic tapered fiber coupling, we demonstrated sensitivity enhancement of two orders of magnitude in dip depth change relative to linewidth change for sensing dye in methanol inside a hollow bottle resonator (HBR) [9].

We recently showed that controlling the nonadiabaticity of an asymmetric tapered fiber allows excitation of desired fiber modes and avoids excitation of undesired modes that do not couple into the microresonator WGM but only provide loss [10]. Three nonadiabatic taper profiles with varying degrees of nonadiabaticity were fabricated and used to provide input to the WGM of an HBR for methane sensing. The degree of nonadiabaticity is determined by the initial taper angle, Ω_2_, of the downtaper, increasing as Ω_2_ increases [10]. Profile 1 (Ω_2_ = 2.50 × 10^−2^) had the lowest nonadiabaticity, profile 2 (Ω_2_ = 3.47 × 10^−2^) the highest and profile 3 (Ω_2_ = 3.00 × 10^−2^) had intermediate nonadiabaticity. Using these fiber taper profiles, we want to demonstrate absorption sensing enhancement like that of Reference [9], but in a narrowband (GHz) absorber.

Some other recent sensing work for comparison is noted here. One involves another microcapillary-based system, but for dispersive sensing [11]. Their method uses the Vernier effect of coupled WGMs (*Q* ~ 8 × 10^3^) to achieve sevenfold enhancement. Another uses self-modulation of a mm-size lithium niobate resonator with WGMs having *Q* ~ 2 × 10^7^ to improve dispersive coupling sensing [12]. Two others deal directly with methane sensing. One uses an on-chip 100-μm ring resonator (*Q* ~ 10^5^) at telecom wavelengths to achieve 153-ppb sensitivity by employing machine learning [13]. The other takes a photoacoustic cell and quartz tuning fork to achieve ppm sensitivity in the mid-infrared [14]. By contrast, our method operates in the telecom band, relies on dip depth changes in a microresonator’s throughput and does not require modulation, frequency locking, or nanofabricated photonic chips. We estimate our detection limit to be hundreds of ppm in a 100-μm resonator having *Q* values around 3 × 10^6^ (details later), somewhat lower than the state-of-the-art values exceeding 10^8^ that have been used in the best WGM sensors [1,2,3,4,5].

Our system is also immune to laser power and temperature fluctuations because it is based on the measurement of changes in *relative* dip depth, independent of power and irrespective of temperature shifts in the WGM resonance frequency (which will be small compared to the methane absorption linewidth in any case). These features make it well-suited for integration into compact, low cost, and potentially field-deployable sensing systems. Our goal is to demonstrate that dip depth-based dissipative sensing with multimode input, achieved via nonadiabatic tapered fiber coupling, is practical for trace gas detection using an HBR. The HBR is fabricated from a silica capillary by heating and pressurizing to form a bottle-shaped bulge [15,16]. Its WGM frequencies can be strain-tuned, by axial stretching, to bring a WGM into resonance with a GHz linewidth gas absorption line for absorption sensing. Because our sensing method uses a resonator fabricated on a capillary, it can also be extended to other analytes in microfluidic environments, beyond what we have already demonstrated [9].

In this paper, we experimentally confirm that dissipative sensing based on dip depth changes can be two orders of magnitude more sensitive than sensing based on linewidth changes when detecting varying concentrations of pure and trace methane. In Section 2, we summarize the theoretical model used to calculate the expected sensitivity enhancement factor, describe the experimental setup, and outline the experimental procedure. We also describe the method for determining the absorption coefficients for both pure and trace methane using the HITRAN database. Section 3 presents the experimental results including dip depth and linewidth responses and confirms that the observed enhancement factors are consistent with theoretical predictions and with the expected values found from the model. In Section 4 we discuss and evaluate the results.

## 2. Materials and Methods

### 2.1. Summary of Theoretical Model

We will follow the treatment that has been described in detail in our earlier works [8,9] and is summarized in the following. We assume that two modes excited in the tapered fiber waist having the same frequency, but different propagation constants and amplitudes, will couple into a single WGM of the HBR having an internal loss *αL*. Sensing is done when the two fiber modes are in phase at the WGM coupling position along the fiber waist. This position is found by translating the fiber along its length relative to the HBR. The ratio of amplitudes of the two fiber modes excited by passage of the light through the nonadiabatic downtaper, higher-order (LP_11_) to fundamental (HE_11_), is denoted by *m*. The incoupling strength and outcoupling loss between the fiber modes and the WGM are assumed to be equal [8] and given by *T*_1_ for the fundamental fiber mode and *T*_2_ for the higher-order fiber mode. The degree of nonadiabaticity of the fiber’s downtaper will determine how strongly various higher-order fiber modes get excited relative to the fundamental mode, and this sets the value of *m*. The fibers to be used are designed to minimize excitation of higher-order fiber modes that do not couple into the WGM and only contribute to additional loss [10].

The relative throughput dip depth as a function of internal loss is given by

(1) $$M\left(\alpha L\right)=\frac{4\left[\left(T_{1}+\sqrt{T_{1}T_{2}}m\right)\left(T_{2}+\alpha L-\sqrt{T_{1}T_{2}}m\right)\right]\;}{{\left(T_{1}+T_{2}+\alpha L\;\right)}^{2}}.$$

Upon introducing an absorbing analyte, the effective internal loss changes, resulting in a fractional change in the dip depth. For our case of an HBR filled with a gaseous analyte the effective internal loss is

(2) $$\alpha L=\alpha_{i}L+f\alpha_{a}L,$$

where *α_i_* is the intrinsic loss coefficient of the microresonator that includes scattering, absorption, and radiation losses, and *α_a_* is the absorption coefficient of the analyte. The microresonator circumference is *L*, and *f* is the internal evanescent (interacting) fraction [17] of the WGM that interacts with the analyte. The change in effective internal loss is due to the addition of the analyte: *dαL* = *fα_a_L*.

We have already shown that, in the limit of small changes *dαL*, the fractional change in dip depth is given by [8]

(3) $$\frac{1}{M}\frac{dM}{d\alpha L}=\frac{1}{T_{1}+T_{2}+\alpha L}\left[\frac{2\sqrt{R_{00}}}{1-\sqrt{R_{00}}}\right],$$

where $R_{00}=1-M(\alpha_{i}L)$ is the relative throughput power in that limit, with the two fiber modes in phase. (With the two fiber modes out of phase when they couple to the WGM, the relative throughput power will be $R_{0\pi }>1$, representing a small peak in the throughput.) To compare the dip depth sensing response to the linewidth sensing response, we begin with the relationship between the linewidth Δ*ν* and the total loss,

(4) $$\Delta \nu =\frac{c}{4\pi^{2}na}\left(T_{1}+T_{2}+\alpha L\;\right),$$

where *c* is the speed of light, *n* is the effective refractive index of the WGM, and *a* is the microresonator radius. From this, we easily find that

(5) $$\frac{1}{\Delta \nu }\frac{d\Delta \nu }{d\alpha L}=\frac{1}{T_{1}+T_{2}+\alpha L},$$

and, therefore, the enhancement of dip depth sensing relative to linewidth sensing is given by

(6) $$\eta =\frac{1}{M}\frac{dM}{d\alpha L}/\frac{1}{\Delta \nu }\frac{d\Delta \nu }{d\alpha L}=\frac{2\sqrt{R_{00}}}{1-\sqrt{R_{00}}}.$$

To calculate the effective absorption path length, we assume an exponential dependence of *M* on *α_a_* and use

(7) $$\frac{dM}{M}=\alpha_{a}L_{eff}=\eta \frac{d\Delta \nu }{\Delta \nu }.$$

Using Equation (5) and the equation for the WGM quality factor, *Q* = *ν*/Δ*ν*, where *ν* is the frequency of the light, and by substituting $d\alpha L=f\alpha_{a}L=2\pi af\alpha_{a}$, we get

(8) $$L_{eff}=\frac{\eta f\lambda Q}{2\pi n},$$

where *λ* is the wavelength of the light. Note that *L_eff_* is proportional to *Q*, whereas *η* is independent of *Q* [8,9]. Equation (3) shows that the fractional change in dip depth also depends on *Q*, and thus so will the limit of detection (LoD); to estimate the LoD, we estimate how small a dip depth change we can reliably measure and multiply that minimum by 3, giving LoDs in the hundreds of ppm.

### 2.2. Experimental Setup and Procedure

**Figure 1:**
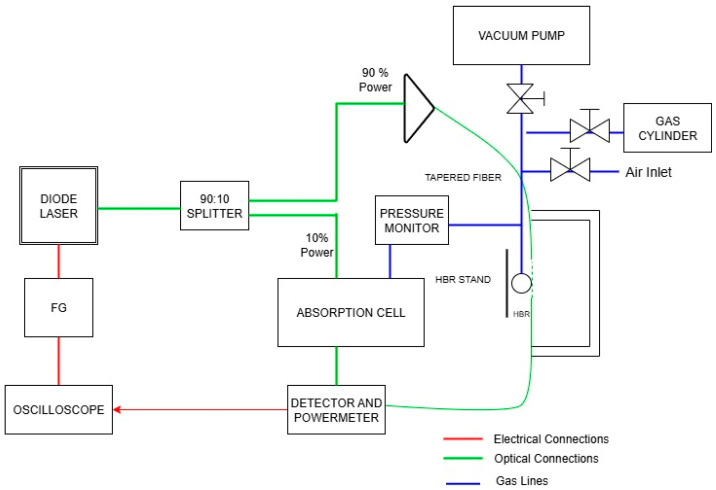
Illustration of the experimental setup.

**Figure 2:**
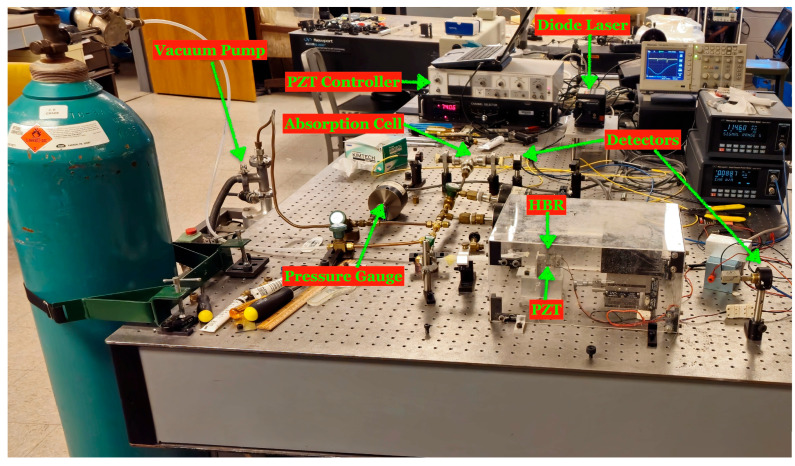
Photograph of the experimental setup.

An illustration of the experimental setup for methane sensing is shown in Figure 1 and a photograph is shown in Figure 2. As a light source we use a tunable diode laser (New Focus, model 6330) with a wavelength range of 1548 nm to 1659 nm. To scan the laser over a 10 GHz frequency range we use a triangular wave from a function generator (WaveTek, model 166). The laser output is sent through a fiber coupler connected to a 90:10 splitter, where 10% of the optical power is directed to a reference absorption cell and 90% is coupled into the tapered fiber. The tapered fiber is used to couple light into and out of WGMs of the HBR, which is sealed at one end.

A 17.5 cm absorption cell is integrated with the vacuum system using copper tubing, while plastic tubing connects the HBR to the vacuum system. In our experiments the pressure and gas mixture are the same in all parts of the system. Three Baratron pressure gauges (10, 100, and 1000 Torr) are used to simultaneously monitor the pressure for better precision in our measurements. The throughput from the tapered fiber and the absorption cell transmission are detected using Newport photodetectors (model 818-IR). The detectors are connected to a power meter, which in turn is connected to a digital oscilloscope (Tektronix, model TDS 2022B). The oscilloscope is triggered using the synchronization output of the function generator, allowing time-aligned measurement with the frequency scan.

In our experiments, the laser wavelength was first tuned to the selected absorption line of methane. The input polarization was set to either TE or TM; the enhancement factor does not depend on polarization [9,10]. Then by coupling light into the HBR’s WGM using one of our asymmetric taper profiles, the dip depth (with fiber modes in phase), linewidth and peak (with fiber modes out of phase) of the WGM of interest were initially recorded with no methane in the HBR. It was verified that the selected WGM is critically coupled when a symmetric fiber, with adiabatic taper transitions and having the same waist radius as the asymmetric tapered fiber, was used to couple light into the WGM [8,9]. Selected concentrations of pure or trace methane were then introduced into the absorption cell and the HBR. The WGM of interest was then strain-tuned into resonance with the gas absorption line. This was done by applying a voltage to the piezoelectric transducer (PZT) to which the HBR was attached, stretching the HBR slightly and shifting the resonance frequency of the WGM.

**Figure 3:**
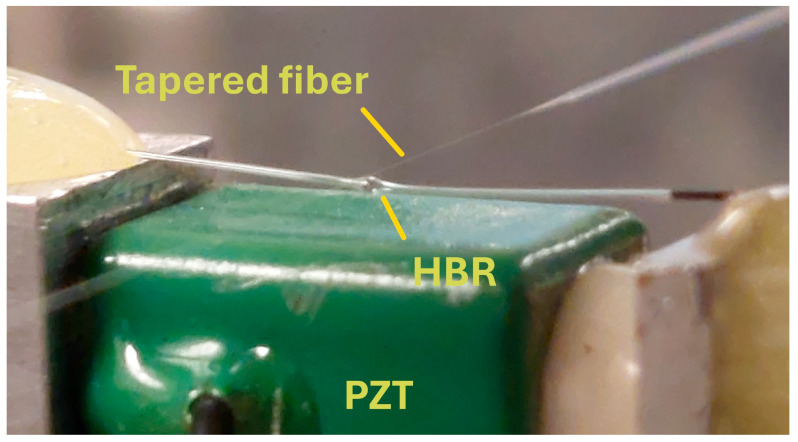
Closeup of the tapered fiber, HBR, and PZT used for strain tuning. An applied voltage expands the PZT, stretching the HBR.

A closeup photo of this part of the setup is shown in Figure 3. Strain tuning of the resonance frequency of the WGM that is doing the sensing is a crucial feature of our experimental procedure. We verified that a WGM could be strain-tuned over a frequency range of 10 GHz, in the absence of methane in the HBR, without changing its linewidth or throughput dip depth. Strain tuning allows us to shift the resonance frequency enough to move the WGM of interest into resonance with the absorption profile or well off-resonance with the absorption profile. This tuning was not affected by any mechanical instabilities or thermal effects and was repeatable to within about 100 MHz.

**Figure 4:**
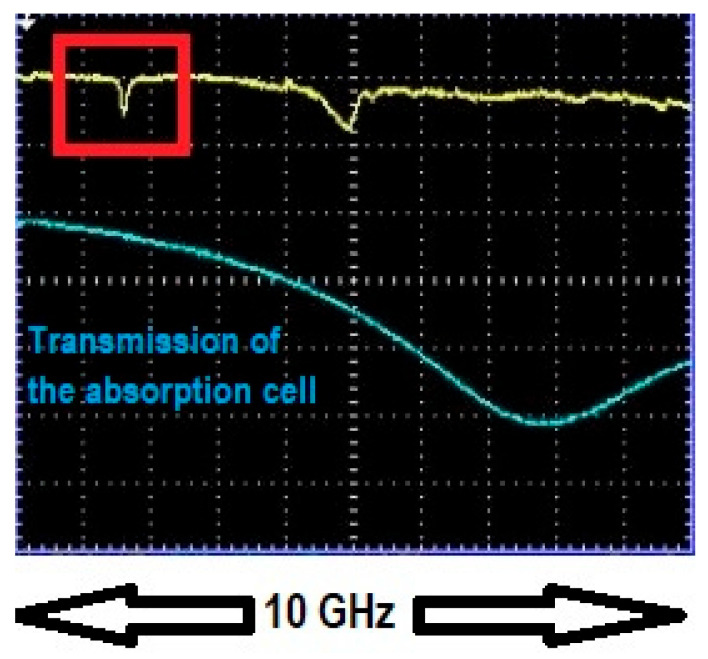
WGM of interest off resonance with 10 Torr trace methane. Channel 1 of the oscilloscope (yellow trace) shows the WGM of interest (in red box) off resonance with the absorption profile. The depth of this dip is *M* = 0.0661, which specifies the vertical scale. Channel 2 (blue trace) shows the intensity transmitted through the absorption cell, used only for locating the position of the absorption line. The absorption cell and HBR are maintained at the same pressure. The wavelength of the laser was 1654 nm.

**Figure 5:**
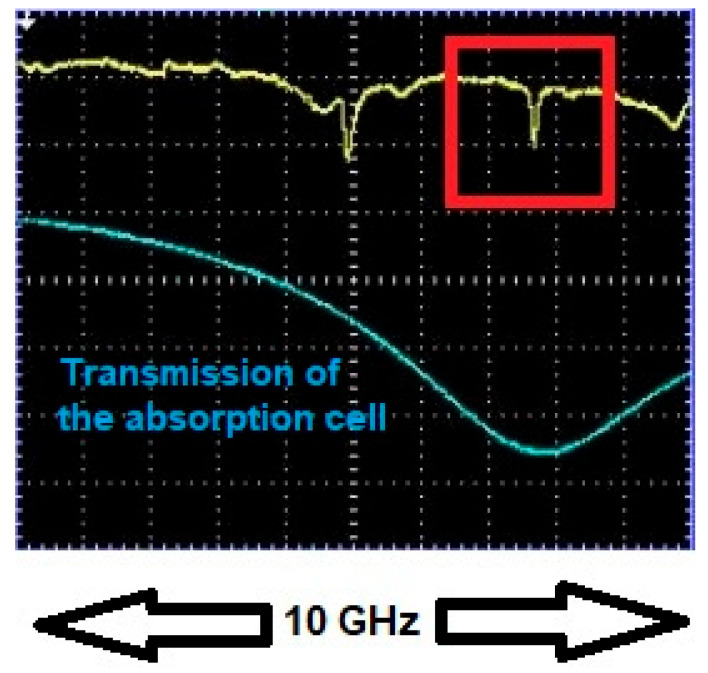
WGM of interest in resonance with 10 Torr trace methane. Channel 1 (yellow trace) shows the WGM of interest (in red box) in resonance with the absorption profile. The dip depth is *M* = 0.108, which specifies the vertical scale. Channel 2 (blue trace) shows the intensity transmitted through the absorption cell, used only for locating the position of the absorption line. The absorption cell and HBR are maintained at the same pressure. The WGM was strain-tuned to bring it into resonance with the absorption profile. The wavelength of the laser was 1654 nm.

Upon achieving resonance, the dip depth and linewidth of the WGM of interest were recorded. Dip depth and linewidth values off resonance were recorded as well and the process was repeated for different gas concentrations. Figure 4 and Figure 5 show one WGM of interest (red rectangular box) when it was in resonance and out of resonance with 10 Torr trace methane (10 Torr of methane in 750 Torr of air). To ensure repeatability all measurements were performed in a randomized order of methane pressures and results for selected concentrations were remeasured two or three times to ensure consistency. Repeated measurements of dip depth and linewidth agreed within their respective uncertainties of ±0.0010 and ±0.22 MHz.

Because our goal is to demonstrate enhancement of the dip-depth response compared to the linewidth response, we want to know the values of *f* and *α_a_*. The methane absorption coefficient *α_a_* can be found from the partial pressure of methane as described in the next paragraph. The interacting fraction *f* can be determined by fitting experimental values of dip depth *M* at various pressures to the formula of Equation (1). At each pressure a value of *f* is found. Those values typically vary within a range of ±5%, and the mean of these is then used. In Equation (1), the values of $T_{1},\;T_{2}+\alpha L,\;\mathrm{and}\;\sqrt{T_{1}T_{2}}m$ can be found from the measurements of *R*_00_ and *R*_0*π*_ [8]. With *f* and *α_a_* known, theoretical values of the dip depth and linewidth can be found from Equations (1) and (4) at each pressure for comparison to the experimentally measured values. These then give theoretical values of the fractional changes in both from Equations (3) and (5) that can be used in Equation (6) for the enhancement factor to find $\eta_{Theory}$ for comparison to $\eta_{Expt}$ and to $\eta_{Model}=2\sqrt{R_{00}}/\left(1-\sqrt{R_{00}}\right)$.

To calculate the absorption coefficients of pure and trace methane at 1654 and 1651 nm, we used parameters including line center positions, line strengths, and pressure broadening coefficients obtained from the HITRAN database [18]. The transition at 1654 nm (1651 nm) consists of three (four) nearby absorption lines, each with its own intensity, linewidth, and lineshape. To obtain the overall absorption spectrum, the contributions from these individual transitions are summed. The linewidth of each transition depends on both Doppler and pressure broadening. The Doppler linewidth at room temperature is 561 MHz for methane at both wavelengths. The pressure broadened linewidth Δ$v_{p}$ for trace methane in air was calculated from the HITRAN database to be 6.2 MHz/Torr at both wavelengths, and the self-broadening of the individual transitions ranged from 4.6 to 6.1 MHz/Torr. The lineshape of each individual transition was found by convolving the Gaussian Doppler profile with the Lorentzian pressure-broadened profile to give a Voigt lineshape, as in Equation (9). The Mathematica code to do this is available upon request.

(9) $$g_{t}\left(\nu -\nu_{0}\right)={\left(\frac{\ln2}{\pi^{3}}\right)}^{1/2}\frac{\Delta \nu_{h}}{\Delta \nu_{i}}\int_{-\infty }^{\infty }\frac{\exp\left[-4\ln2{\left(\frac{\nu_{0}^{\prime }-\nu_{0}}{\Delta \nu_{i}}\right)}^{2}\right]}{{\left(\nu -\nu_{0}^{\prime }\right)}^{2}+{\left(\frac{\Delta \nu_{h}}{2}\right)}^{2}}d\nu_{0}^{\prime }$$

In Equation (9), *ν*_0_ denotes line center, Δ*ν_h_* is the homogeneous linewidth incorporating both self- and air-pressure broadening, and Δ*ν_i_* is the Doppler linewidth.

**Figure 6:**
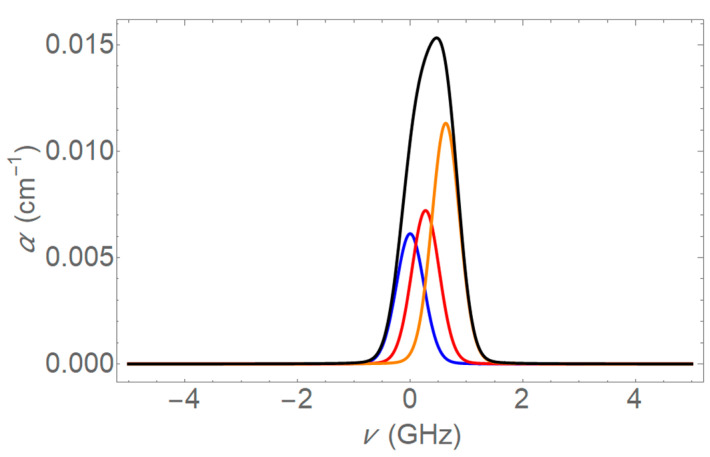
Absorption profile for 5 Torr pure methane at 1654 nm. This graph shows the absorption coefficient as a function of frequency detuning (in GHz). The blue, red, and yellow curves represent the individual transitions of methane, and the black curve represents the overall absorption profile obtained by combining the individual transitions.

**Figure 7:**
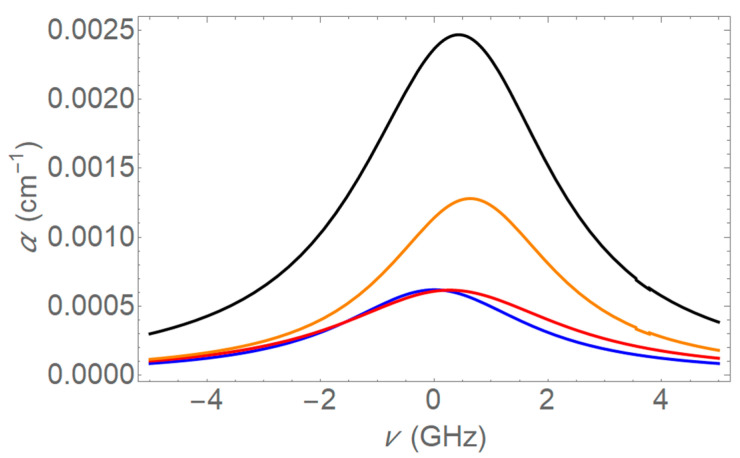
Absorption profile for 5 Torr methane in 755 Torr air at 1654 nm. This plot shows the absorption coefficient as a function of frequency detuning (in GHz). The red, yellow, and blue curves represent the individual pressure broadened lines and the overall absorption profile is shown in black. Pressure broadening is dominant, resulting in broader absorption profiles than in Figure 6.

The absorption profiles at 1654 nm for 5 Torr pure methane and 5 Torr trace methane are given in Figure 6 and Figure 7. Similar profiles can be found by combining the four transitions at 1651 nm.

**Figure 8:**
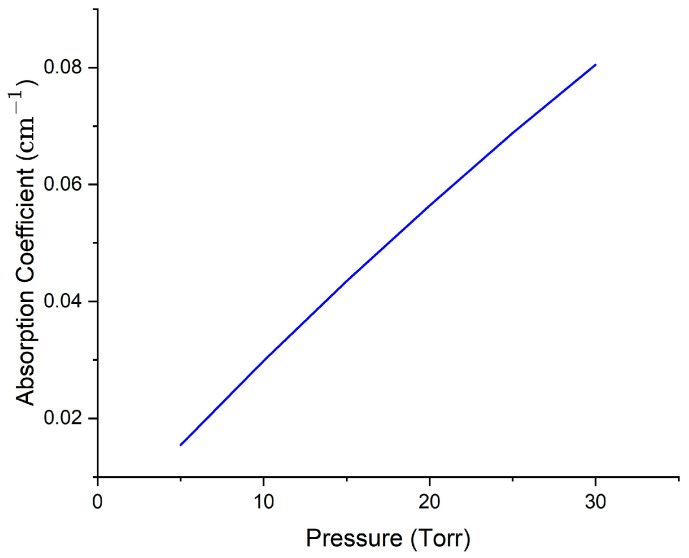
Plot of peak absorption coefficient vs. pressure for pure methane at 1654 nm. Data from HITRAN were used to do the convolution via Mathematica programming to get the overall absorption profile and peak absorption coefficient, such as the maximum of the black curve in Figure 6, at each pressure.

**Figure 9:**
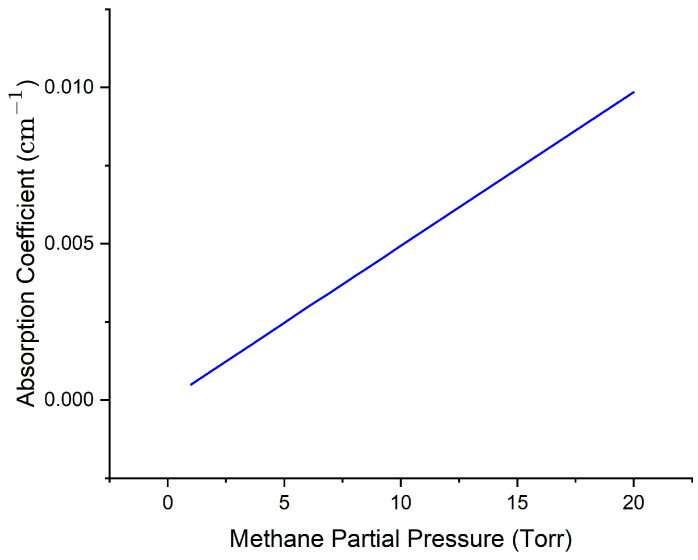
Plot of peak absorption coefficient vs. pressure for trace methane at 1654 nm. Methane and air partial pressures add to 1 atm. Data from HITRAN were used to do the convolution via Mathematica programming to get the overall absorption profile and peak absorption coefficient, such as the maximum of the black curve in Figure 7, at each methane partial pressure.

Peak absorption coefficients were calculated for pure and trace methane at 1654 nm and are plotted in Figure 8 and Figure 9. It can be noted that the pressure ranges plotted in these two figures are like those used in actual experiments for both pure and trace gas conditions. The plots show a near-linear relationship; nonlinear behavior will only become significant at higher pressures, beyond the experimental range. Since the primary goal is to examine sensing behavior at low concentrations, all calculations involving dip depth/linewidth slope ratios and comparative analyses are performed in this linear region, ensuring that the assumptions made in our model remain valid.

## 3. Experimental Results

The experimental procedure described in Section 2.2 was carried out for several WGMs in several HBRs using three asymmetric tapered fibers having taper profiles with different nonadiabaticities, each tapered down to a waist radius of 1.47 µm [10]. In most cases, the HBR used had an outer radius in the range of 90–100 µm and was fabricated by heating and pressurizing a fused silica capillary to form a bottle-shaped bulge. The waist radius of 1.47 µm was specifically chosen because, for microresonators of this size, it results in approximately equal coupling of the WGM to the fundamental HE_11_ fiber waist mode and the first higher order LP_11_ mode, while coupling to higher-order modes beyond LP_11_ remains significantly weaker [19].

The analysis of each experimental case involves the following steps. First the interacting fraction *f* of the WGM of interest is determined as described in Section 2.2 and used to calculate the theoretical values of dip depth and linewidth at each pressure, enabling direct comparison with the experimental values. The fractional change in dip depth is defined as

(10) $$\frac{\Delta M}{M}=\frac{M\left(\alpha L\right)-M\left(\alpha_{i}L\right)}{M\left(\alpha_{i}L\right)},$$

where *M*(*αL*) is the dip depth on resonance at the methane concentration or partial pressure of interest and *M*(*α_i_L*) is the dip depth when the WGM is detuned far off resonance to minimize methane absorption. This fractional change data point then has error bars that are calculated from the uncertainty of ±0.0010 in each *M*. The fractional change in linewidth is found in a similar manner, with error bars calculated from the uncertainty of ±0.22 MHz in each linewidth. These errors are propagated as described below to find the uncertainty in the enhancement factor. Theoretical and experimental fractional changes in dip depth are plotted against gas concentration and their respective slopes and slope uncertainties are determined using Origin software. Slope uncertainties are determined from the individual error bars at each pressure and from the scatter of the points about the straight line. The same is done for theoretical and experimental fractional changes in linewidth. Theoretical and experimental enhancement factors are then calculated as the ratio of the respective slopes of dip depth change and linewidth change and compared with the model predicted enhancement factor as in Equation (6).

### 3.1. Pure Methane Sensing

**Figure 10:**
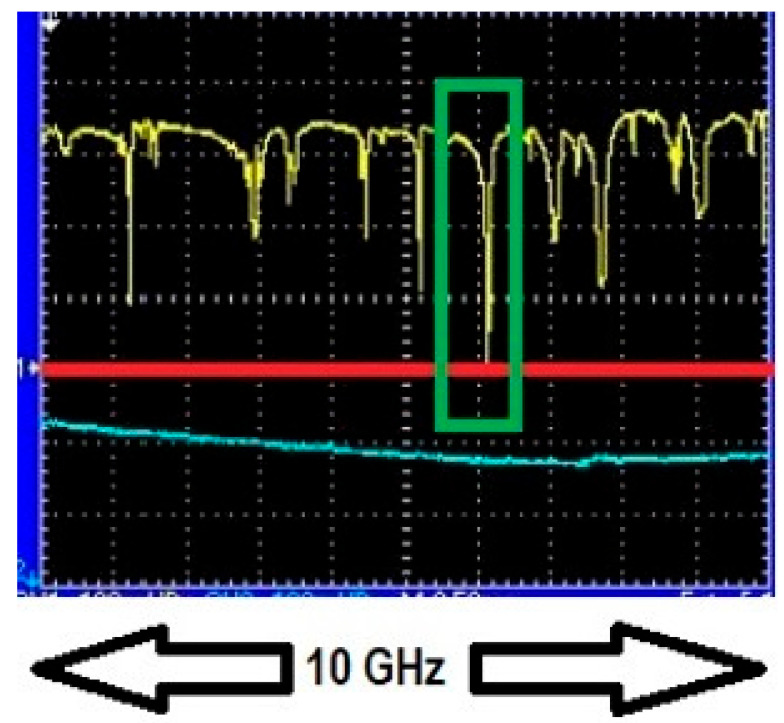
Critical coupling observed when a symmetric fiber of the same waist radius as the asymmetric fiber was used to couple light into the HBR. The throughput dip of the WGM of interest seen in channel 1 (highlighted in a green box) can be seen reaching the zero-voltage baseline (red line). This means that *M* = 1.0, indicating critical coupling. There was no methane present in the experimental setup as can be seen by the lack of any feature in the absorption cell transmission in channel 2 (blue trace).

**Figure 11:**
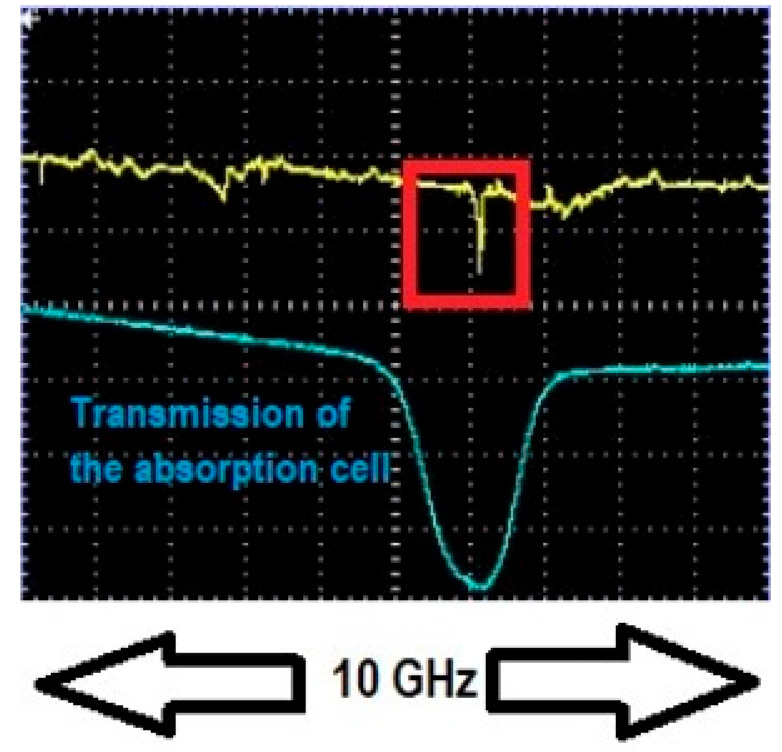
WGM of interest (highlighted in a red box) in resonance with the absorption line at 25 Torr methane concentration. The dip depth is *M* = 0.212, which specifies the vertical scale. Channel 2 (blue trace) shows the transmission through the 17.5 cm absorption cell, used only for locating the position of the absorption line, at 1654 nm laser wavelength.

**Figure 12:**
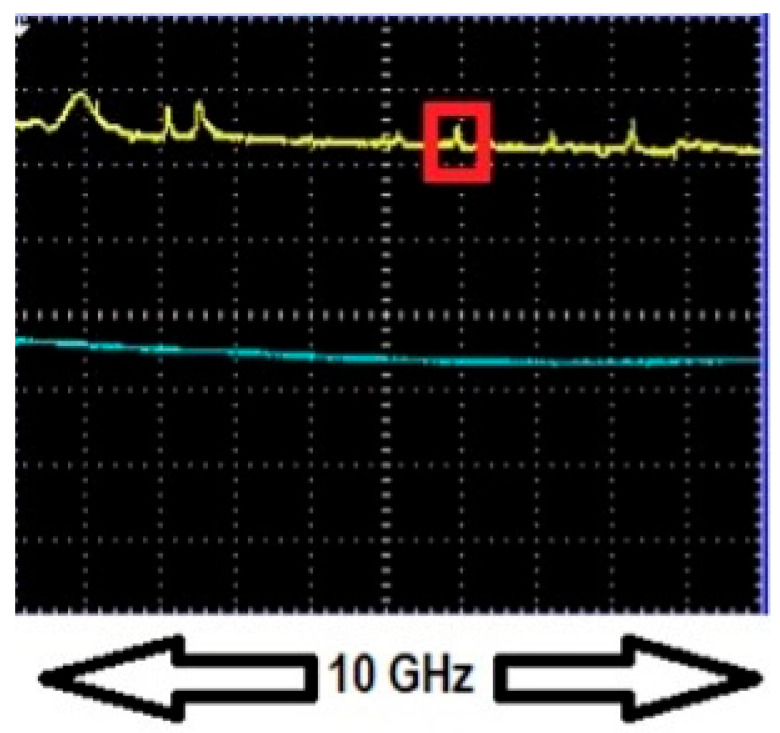
Peaks when fiber modes are out of phase observed in channel 1 (yellow trace) by translating the point of contact between the HBR and the asymmetrically tapered fiber. The peak corresponding to the WGM of interest is highlighted in a red box. For this WGM, the relative throughput is *R*_0π_ = 1.038, which specifies the vertical scale. There was no methane present in the experimental setup as can be seen by the lack of any feature in the absorption cell transmission in channel 2 (blue trace).

We begin with experiments using low-pressure (25 Torr or less) pure methane to confirm that our enhancement method can be applied to a narrowband (GHz) absorber. Analysis of one such experiment using taper profile 1 at 1654 nm is presented. The HBR used had a radius of 90 µm. The parameters of the WGM of interest, namely dip depth, linewidth, and peak, were recorded when no methane was present in the experimental setup. An adiabatic fiber of the same waist radius as the nonadiabatic fiber was used to ensure that the WGM is critically coupled, as shown in Figure 10. The WGM of interest was tuned by applying a voltage to the PZT to be in resonance or out of resonance with the absorption profile at different pressures of methane and its dip depth and linewidth were recorded. Figure 11 shows the WGM of interest in resonance with 25 Torr pure methane at 1654 nm and Figure 12 shows peaks observed by translating the point of coupling between the fiber modes and the WGM along the fiber waist until the fiber modes were out of phase.

**Figure 13:**
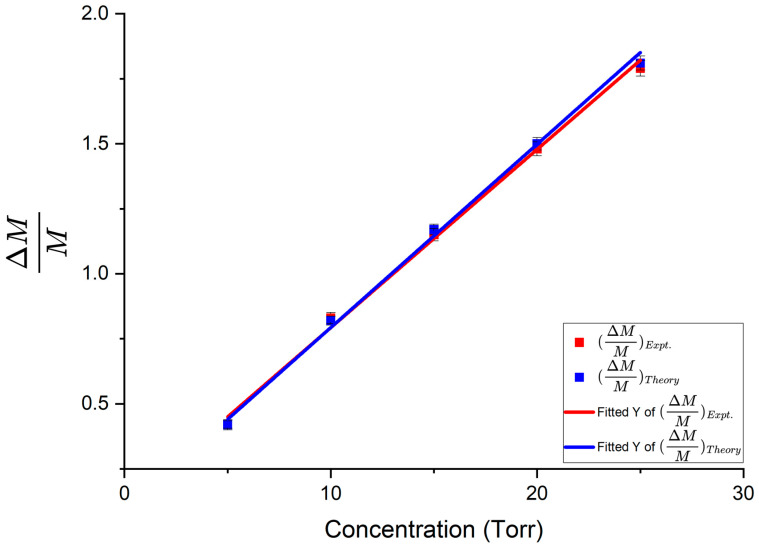
Fractional change in dip depth as a function of methane pressure. Asymmetric taper profile 1 was used to couple light into and out of the HBR. The red line represents the experimental fractional change in dip depth and the blue line represents the theoretical fractional change in dip depth. Error bars are shown on both experimental and theoretical points.

The following measurements were recorded when no methane was present in the system. Dip depth *M* = 0.0758, linewidth Δ*ν* = 65.50 MHz, *R*_00_ = 0.9242, *R*_0π_ = 1.05. Upon introducing methane of increasing pressure into the experimental setup, the dip depth and linewidth increased. Dip depths corresponding to different gas concentrations were recorded and fitted to the model with the interacting fraction *f* of the WGM being the fitting parameter. The mean *f* for this WGM was 0.0113 and this value was used in all the subsequent calculations. Fractional change in dip depth is plotted as a function of methane pressure in Figure 13. The slopes of best fit found using Origin software were 0.07052 ± 0.00127/Torr for the theoretical data points and 0.06857 ± 0.00155/Torr for the experimental data points.

**Figure 14:**
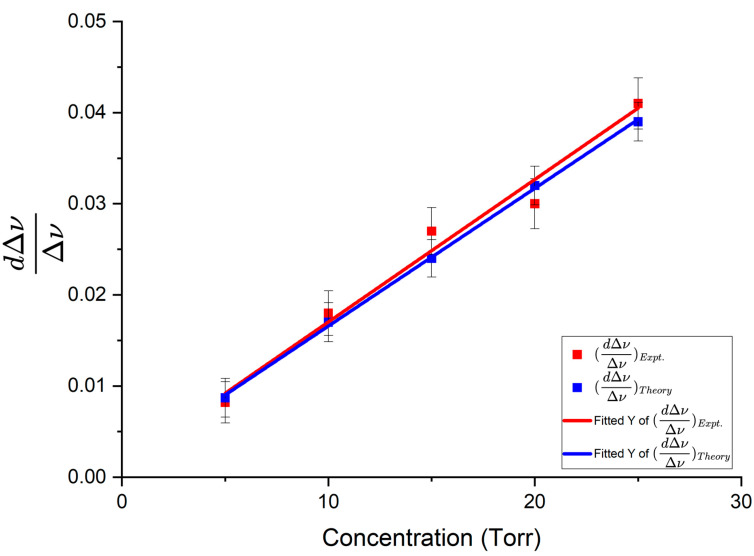
Fractional change in linewidth as a function of methane pressure. Asymmetric taper profile 1 was used to couple light into and out of the HBR. The red line represents the experimental fractional change in linewidth and the blue line represents the theoretical fractional change in linewidth. Error bars are shown on both experimental and theoretical points.

Fractional change in linewidth is plotted as a function of methane pressure in Figure 14. The slopes of best fit were 0.00151 ± 0.00013/Torr for theoretical data points and 0.00156 ± 0.00016/Torr for experimental datapoints.

Using the experimental and theoretical slopes from the graphs in Figure 13 and Figure 14, the experimental enhancement was *η_Expt_* = 43.95 ± 4.64 and the theoretical enhancement was *η_Theory_* = 46.70 ± 4.22. The enhancement factor predicted by the model was *η_Model_* = 49.75 ± 0.67. From these results, we see that the value of the enhancement factor obtained from the theoretical slopes is within the uncertainty limits of the enhancement factor predicted by the model; the experimental enhancement is the smallest but reasonably close to the model predicted value. A summary of the experimental results obtained using the three taper profiles, following similar analyses to that presented above, is given in Table 1.

We see that in all cases, the enhancement factors are nearly two orders of magnitude. Those predicted by our model and those calculated from the theoretical slopes are in good agreement within their uncertainty limits. The enhancement factors obtained from the experimental slopes are usually slightly lower, but still reasonably close. With this confirmation of the applicability of our enhancement method to a narrowband absorber, we next go to the more realistic situation of trace amounts of methane in one atmosphere of air.

### 3.2. Trace Methane Sensing

**Figure 15:**
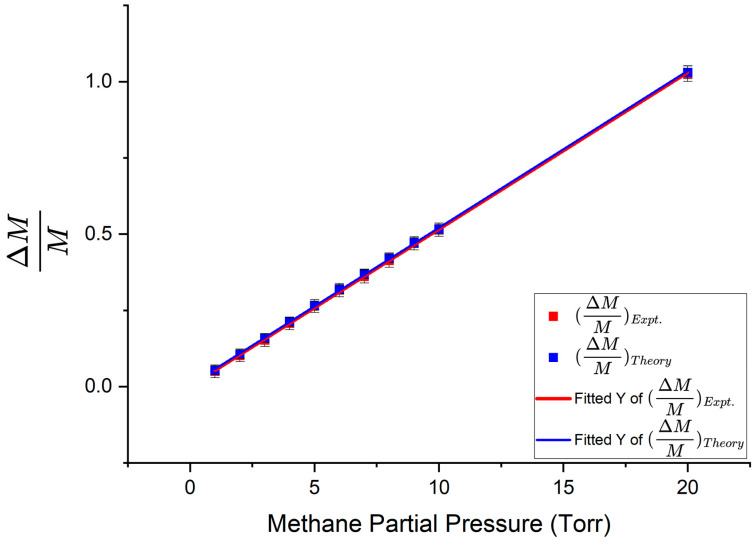
Fractional change in dip depth as a function of methane partial pressure. Asymmetric taper profile 3 was used to couple light into and out of the HBR. The red line represents the experimental fractional change in dip depth and the blue line represents the theoretical fractional change in dip depth. Error bars are shown on both experimental and theoretical points.

Experiments for trace methane sensing were performed by allowing a certain concentration of methane into the experimental setup (both the absorption cell and HBR) and then allowing air in until the pressure monitor showed a reading of 760 Torr. Dip depths and linewidths of the WGM of interest were recorded in and out of resonance with the absorption line by strain-tuning the HBR. After recording the measurements, the setup was pumped out and the process was repeated for other mixtures of methane and air. For the WGM at 1654 nm discussed below, the HBR had a radius of 100 µm and taper profile 3 was used to couple light into and out of it. The following measurements were recorded when no methane was present in the system. Dip depth *M* = 0.0679, linewidth Δ*ν* = 85.48 MHz, *R*_00_ = 0.9321, *R*_0π_ = 1.043. When methane of increasing partial pressures was introduced into the experimental setup, dip depths and linewidths increased. Dip depths corresponding to different methane partial pressures were recorded and fitted to the model with the interacting fraction *f* of the WGM being the fitting parameter. The interacting fraction of this WGM was found to be 0.0502 by averaging all the fitting parameters and this value was used in all subsequent calculations. Fractional change in dip depth is plotted as a function of methane partial pressure in Figure 15. The slopes of best fit found using Origin software were 0.05148 ± 0.00111/Torr for the theoretical data points and 0.05144 ± 0.00143/Torr for the experimental data points.

**Figure 16:**
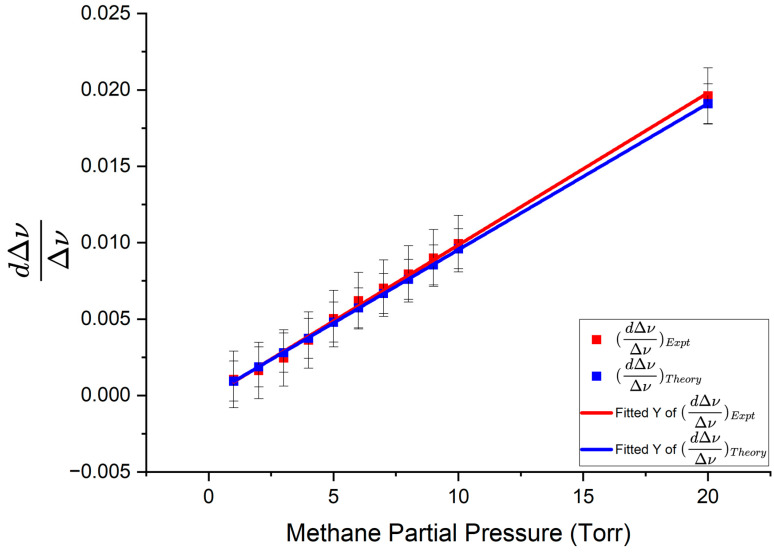
Fractional change in linewidth as a function of methane partial pressure. Asymmetric taper profile 3 was used to couple light into and out of the HBR. The red line represents the experimental fractional change in linewidth and the blue line represents the theoretical fractional change in linewidth. Error bars are shown on both experimental and theoretical points.

Fractional change in linewidth is plotted as a function of methane concentration in Figure 16. The slopes of best fit were 0.000957 ± 0.000079/Torr for the theoretical data points and 0.000996 ± 0.000111/Torr for the experimental data points.

Using the experimental and theoretical slopes from the graphs in Figure 15 and Figure 16, the experimental enhancement was *η_Expt_* = 51.64 ± 5.95 and the theoretical enhancement was *η_Theory_* = 53.75 ± 4.56. The enhancement factor predicted by the model was *η_Model_* = 55.89 ± 0.83. From these results, we see that the values of all three enhancement factors agree within their uncertainty limits, with the experimental enhancement being the smallest. A summary of the experimental results obtained using the three taper profiles, following similar analyses to that presented above, is given in Table 2. Table 2 also gives the interacting fraction *f*, quality factor *Q*, and effective absorption path length *L_eff_* for each case.

From Table 2, it can be observed that in most cases, the enhancement factors predicted by the model and those obtained from the theoretical slope ratios agree well and lie within the stated uncertainty limits. The experimentally determined enhancement factors are typically slightly lower than the other two but remain reasonably close, resulting in a consistent overall agreement among the three approaches that the enhancement is approximately two orders of magnitude. It was observed that the theoretical dip depths were usually higher than the experimental ones, especially for trace gas sensing where getting perfectly off resonance was more difficult. This affected the measured off resonance dip depths more strongly than the linewidths and led to higher estimated off resonance dip depth values, as will be discussed in the next Section. As a result, experimental enhancement factors tended to be lower than those predicted by the model. Keep in mind that, while the enhancement factors are independent of the *Q* of the WGM [8,9], *L_eff_* depends on *Q* (and on *f*; see Equation (8)). The values of *L_eff_* in Table 2 are found from using *η_Expt_* in Equation (8). The values of *f* and *Q* have relative uncertainties of less than 1%, so the percent uncertainty in *L_eff_* is essentially the same as that of *η_Expt_*.

## 4. Discussion and Conclusions

We demonstrated enhancement of dissipative sensing of methane using nonadiabatic tapered fiber-coupled whispering-gallery-mode microresonators. Three tapered fibers with varying nonadiabaticities but the same waist radius of 1.47 µm [10] were fabricated to selectively excite the LP_11_ mode while suppressing unwanted higher-order modes that contribute to loss. Methane absorption at 1654 nm and 1651 nm was modeled using HITRAN data, incorporating both Doppler and pressure broadening via Voigt profiles. Effective absorption path lengths between 101 cm and 273 cm were achieved, with estimated detection limits in the hundreds of ppm. The experimental sensitivity through dip depth changes was found to be up to two orders of magnitude larger than the sensitivity based on linewidth changes, consistent with theoretical predictions. Among the three taper profiles, profile 3, with intermediate nonadiabaticity [10], yielded the highest enhancement factor and the best percentage agreement with the model, demonstrating its superior performance, as summarized in Table 3. The averages in Table 3 are taken over several different WGMs in different HBRs, using both pure and trace methane data.

The enhancement factors predicted by the model were consistently the highest, while those calculated from the theoretical slope values were within the uncertainty range of the model values. The experimental enhancement factors, although slightly lower, were still reasonably close, showing overall consistency among the three methods. The model value is expected to be the largest because it represents the enhancement in the limit as pressure approaches zero, where the dip depth response to pressure is steepest due to slight nonlinearity, while the linewidth response remains more linear across a broader range of pressures. Since both theoretical and experimental enhancement factors are obtained from slope ratios over finite pressure intervals rather than strictly in the *p* → 0 regime, their values tend to fall slightly below the model prediction. This is consistent with the nature of the dip depth curve, which is most sensitive at low pressures but gradually flattens at pressures somewhat higher than those reported in Figure 13 and Figure 15. A recurring trend observed is that the theoretical slope for relative dip depth change is generally larger than the experimental slope, whereas the experimental slope for relative linewidth change is often slightly larger than the theoretical one. This was especially evident in trace gas sensing, where the broader absorption profiles made it harder to get completely off resonance. This led to higher off-resonance dip depth values *M*(*α_i_L*), which in turn reduced the measured fractional dip depth changes according to Equation (10). In addition, the overestimate of *M*(*α_i_L*) in the denominator of Equation (10) led to slopes that were smaller by 2–12%. As the enhancement factor is calculated as a ratio of the dip depth to linewidth slopes, this combination naturally led to lower experimental enhancement factors. As has been shown in previous work involving dye sensing in methanol [9], it was found that the enhancement is independent of quality factor (see also Table 2). Higher *Q* values such as 10^8^ [1,2,3,4,5] would improve the effective path lengths but our results for these were still significant. Even with a *Q* less than 3 × 10^6^, *L_eff_* was greater than 10^4^ times the HBR diameter in one case. Measurements taken in randomized order confirmed repeatability; returning to previously measured conditions yielded experimental results within the measurement error. Faster response times (a few seconds) were observed at low pressures, making this method ideal for detecting small gas concentrations quickly.

While other methods can offer better overall sensitivity, our goal was not to try to compete with them but rather to demonstrate that the enhancement resulting from multimode input can successfully be applied to narrow gas absorption profiles. With the enhancement factors that we found, our method of absorption sensing of methane in the telecom wavelength regime produced sensitivities nearly equivalent to those of linewidth or ringdown absorption sensing in centimeter-length cells in the mid-infrared, where the absorption strengths are two orders of magnitude greater. Our method could also, for example, enable incorporation into a microfluidic gas transport system.

## Figures and Tables

**Table 1:** Summary of pure methane sensing enhancement results.

Taper Profile	λ (nm)	$\eta_{Expt}$	$\eta_{Theory}$	$\eta_{Model}$
1 ^a^	1651	59.20 ± 7.47	65.31 ± 6.53	70.24 ± 1.30
1	1651	38.95 ± 4.37	45.27 ± 4.19	50.03 ± 0.67
1	1654	56.90 ± 6.59	57.81 ± 5.28	63.43 ± 1.07
1	1654	43.95 ± 4.64	46.70 ± 4.22	49.75 ± 0.67
2	1651	42.53 ± 2.68	41.31 ± 2.49	44.65 ± 0.54
2	1654	64.40 ± 6.32	67.83 ± 5.17	75.26 ± 1.49
2 ^a^	1654	56.88 ± 3.52	59.02 ± 2.72	62.66 ± 1.04
3	1651	76.89 ± 9.32	82.12 ± 7.53	88.10 ± 2.03
3	1654	70.08 ± 5.37	74.63 ± 6.38	78.78 ± 1.63
3	1654	42.60 ± 4.74	45.18 ± 3.68	48.72 ± 0.64

^a^ HBR radius 175 µm.

**Table 2:** Summary of trace methane sensing enhancement results. Values of interacting fraction *f*, quality factor *Q*, and effective absorption path length *L_eff_* are also shown.

Taper Profile	λ (nm)	$\eta_{Expt}$	$\eta_{Theory}$	$\eta_{Model}$	*f* (10^−2^)	*Q* (10^6^)	*L_eff_* (cm)
1	1651	64.73 ± 7.72	70.63 ± 6.95	73.91 ± 1.44	3.61	3.90	166
1	1651	53.88 ± 6.53	56.83 ± 5.03	60.07 ± 0.96	3.98	3.50	137
1 ^b^	1654	51.87 ± 7.99	61.11 ± 8.38	64.66 ± 1.11	3.27	4.20	130
1	1654	44.47 ± 4.33	48.18 ± 4.31	51.18 ± 0.70	5.17	2.76	116
2	1651	52.80 ± 4.10	54.38 ± 3.14	58.33 ± 0.91	5.23	3.59	181
2 ^a^	1651	45.90 ± 3.42	48.31 ± 2.63	50.10 ± 0.67	6.14	2.17	112
2	1651	49.82 ± 4.97	50.90 ± 3.27	55.63 ± 0.83	4.26	6.01	233
2	1654	59.82 ± 4.89	60.33 ± 3.64	63.87 ± 1.08	7.40	2.27	183
2	1654	47.97 ± 2.55	48.38 ± 2.45	50.89 ± 0.70	8.11	1.58	113
3	1651	88.97 ± 11.10	93.85 ± 8.76	97.74 ± 2.48	4.08	2.99	198
3	1651	141.19 ± 19.79	144.82 ± 14.84	150.2 5 ± 5.79	3.68	2.88	273
3 ^a^	1651	84.31 ± 13.13	86.32 ± 9.75	91.77 ± 2.19	3.28	5.39	272
3	1654	51.64 ± 5.95	53.75 ± 4.56	55.89 ± 0.83	5.02	2.12	101
3	1654	62.73 ± 4.97	64.90 ± 5.30	67.65 ± 1.21	5.60	2.25	145

^a^ HBR radius 175 µm ^b^ HBR radius 150 µm.

**Table 3:** Average values of enhancement factors for the three taper profiles. The profiles are characterized by number and initial taper angle Ω_2_.

Taper Profile	Ω_2_	$\eta_{Expt}$	$\eta_{Model}$
1	2.50 × 10^−2^	51.74 ± 2.25	60.41 ± 0.36
2	3.47 × 10^−2^	52.52 ± 1.50	57.67 ± 0.34
3	3.00 × 10^−2^	77.30 ± 3.72	84.86 ± 0.92

## Data Availability

Data are available from the corresponding author upon request.

## References

[1] Bozzola A., Perotto S., Angelis F.D. (2017). Hybrid plasmonic–photonic whispering gallery mode resonators for sensing: A critical review. Analyst.

[2] Steglich P., Rabus D.G., Sada C., Paul M., Weller M.G., Mai C., Mai A. (2022). Silicon Photonic Micro-Ring Resonators for Chemical and Biological Sensing: A Tutorial. IEEE Sens. J..

[3] Foreman M.R., Swaim J.D., Vollmer F. (2015). Whispering gallery mode sensors. Adv. Opt. Photonics.

[4] Ward J.M., Dhasmana N., Nic Chormaic S. (2014). Hollow core, whispering gallery resonator sensors. Eur. Phys. J. Spec. Top..

[5] Jiang X., Qavi A.J., Huang S.H., Yang L. (2020). Whispering Gallery Sensors. Matter.

[6] Rosenberger A.T. (2007). Analysis of whispering-gallery microcavity-enhanced chemical absorption sensors. Opt. Express.

[7] Farca G., Shopova S.I., Rosenberger A.T. (2007). Cavity-enhanced laser absorption spectroscopy using microresonator whispering-gallery modes. Opt. Express.

[8] Rajagopal S.R., Rosenberger A.T. (2022). Enhancement of Dissipative Sensing in a Microresonator Using Multimode Input. Sensors.

[9] Rajagopal S.R., Ke L., Sandoval K., Rosenberger A.T. (2023). Confirmation of Dissipative Sensing Enhancement in a Microresonator Using Multimode Input. Sensors.

[10] Rosenberger A.T., Haq M.J.U. (2025). Design of Asymmetrically Tapered Fibers for Multimode Input to Enable Dissipative Sensing Enhancement in Microresonators.

[11] Li B., Liu L., Xu L. (2025). Ultra-sensitive active whispering gallery mode sensor in an optofluidic microcapillary with Vernier effect of coupled modes. Opt. Express.

[12] Jia T., Xing E., Li J., Rong J., Yue H., Zhang Y., Xing G., Zhou Y., Liu W., Tang J. (2025). High-precision quasi-static sensing method based on WGM resonator self-modulation. Photonics Res..

[13] Qin P., Kang X., Gan X. (2025). Machine learning-enhanced on-chip micro-ring resonator platform for detection and recognition of low-concentration gas mixtures. Opt. Express.

[14] Angstenberger S., Floess M., Schmid L., Ruchka P., Steinle T., Giessen H. (2025). Coherent control in quartz-enhanced photoacoustics: Fingerprinting a trace gas at ppm-level within seconds. Optica.

[15] Stoian R.-I., Bui K.V., Rosenberger A.T. (2015). Silica hollow bottle resonators for use as whispering gallery mode based chemical sensors. J. Opt..

[16] Murugan G.S., Petrovich M.N., Jung Y., Wilkinson J.S., Zervas M.N. (2011). Hollow-bottle optical microresonators. Opt. Express.

[17] Consani C., Dubois F., Auböck G. (2021). Figures of merit for mid-IR evanescent-wave absorption sensors and their simulation by FEM methods. Opt. Express.

[18] Gordon I.E., Rothman L.S., Hargreaves R.J., Hashemi R., Karlovets E.V., Skinner F.M., Conway E., Hill C., Kochanov R., Tan Y. (2022). The Hitran 2020 molecular spectroscopic database. J. Quant. Spectrosc. Radiat. Transf..

[19] Humphrey M.J., Dale E., Rosenberger A.T., Bandy D.K. (2007). Calculation of optimal fiber radius and whispering-gallery mode spectra for a fiber-coupled microsphere. Opt. Commun..

